# The Southern Surgical and Gynecological Association

**Published:** 1904-01

**Authors:** 


### THE SOUTHERN SURGICAL AND GYNECOLOGICAL ASSOCIATION.

THE Southern Surgical and Gynecological Association, which convened in Atlanta during the month of December, for three days, was very largely attended. It was very much enjoyed by the physicians of Atlanta and neighboring States, as well as by those who came from all parts of the country.

It was one of the most representative gatherings that has taken place since this Association was founded by Dr. W. E. B. Davis, in his office in Birmingham, and that he was not present was due to the hand of death, which has been busy during the past year, taking the young as well as the old.

This meeting was the fitting occasion for the action of the Association and the profession for the erection of a monument to his memory, in the city of Birmingham, Ala., where the Association will meet next year.

We were not fortunate enough to hear all of the papers, but heard good words of comment upon them.

The memorial address by Dr. Richard Douglas upon Dr. W. E. B. Davis was listened to with marked attention, as was also the presidential address by Dr. J. W. Bovee.

Among the impressive personalities on the programme were Drs. W. B. Coley, of New York; M. H. Richardson, of Boston; Dr. C. H. Mayo, of Rochester, Minn., and Dr. J. Horace Whitacre, of Cincinnati. In the election of Dr. Floyd W. McRae, of Atlanta, as the next president, we feel honored.

Dr. C. N. Chavigny, of New Orleans, though one of the youngest members, interested the Associatiom greatly in his account of "Pus Collections in Pelvis."

The paper of Dr. J. Riddle Goffe, of New York, was also very edifying to those who heard it, although it brought out a lively discussion, partaking of criticism in which many seemed to regard the vaginal route a more difficult one than the abdominal route in operating for pelvic diseases; however, it can be stated that Dr. Goffe has had very excellent results and has succeeded admirably in the work which he has undertaken to do. We suppose that any one who has given his attention to this operation will become more expert in the performance than one who has been using abdominal incision. We presume that both have very special advantages and that success is in the choice of treatment; so that the surgeon who uses his judgment before operating does best.

It is also worthy of note that some surgeons who have adopted the vaginal route have found it practicable and have sanctioned it by their reports.

From the number of papers on the subject of appendicitis, which also enlisted the attention and brought out the views of those present, much information was gained. The paper of Dr. Dean, of Spartanburg, advised operation in every case; and, as the writer has said before, each case should be considered carefully and prayerfully before operation.

There were several papers on the gall-bladder and the surgery best adapted to the treatment of this sac and its ducts.

The discussion of the paper of Dr. Mack Rogers, of Birmingham, was quite interesting, and showed a great diversity of opinion on "gall-stones."

Some of the papers were not read in the order in which they were arranged on the programme, due to the absence of many of those who intended to come, but the programme was an especially full one, and many of the papers were read and discussed.

Dr. Crile's paper was especially interesting, as he exhibited the rubber suit which was used as the means of treating blood pressure, and his remarks upon the use of adrenaline in shock were instructive.

The paper of Dr. A. A. Shands, of Washington, was a useful presentation of the subject and gave rise to some interesting comment by Dr. Nicolson, of Atlanta, with his experience with crinoline.

The papers of Dr. Balloch, of Washington, and Dr. Gaston, of

Atlanta, treated of some cases in genito-urinary surgery and the discussion they received was encouraging.

Another case of interest reported by an Atlanta member was that of sarcoma of the psoas magnus muscle, by Dr. George H. Noble.

To sum up the papers, they covered all the new facts of surgery and gynecology, and the Transactions of the Association will be looked forward to with interest.

The members of the Association and some of the local profession were delightfully entertained at a reception tendered by Dr. Willis F. Westmoreland, at Brookwood, followed on the next night by a large reception at the Capital City Club. Much credit is due to the local committee of arrangements for their untiring efforts for the entertainment of the Association members.

We are just in receipt of an invitation from the Hunter McGuire Memorial Association to the unveiling of the statue to the memory of Hunter McGuire, M.D., LL.D., Thursday afternoon at one o'clock, January 7, 1904, at Capitol Square, Richmond, Va., and it is very gratifying to those who have watched the progress of surgery in the South to note the success which has attended the labors of these surgeons who have been true to their Southland. The Southern Surgical and Gynecological Association is a monument more lasting than bronze to the memory of these surgeons as well as many others who have contributed to the Transactions of the Association. A complete set of the published volumes of the Transactions of the Association will compare favorably with the work done by the American Surgical Association, or any other surgical association.

